# Decomposition characteristics of indigenous organic fertilisers and introduced quick compost and their short-term nitrogen availability in the semi-arid Ethiopian Rift Valley

**DOI:** 10.1038/s41598-019-52497-8

**Published:** 2019-11-05

**Authors:** Shiro Mukai, Wataru Oyanagi

**Affiliations:** 1 1-19-7 Omorinishi, Ota-ku, Tokyo 143-0015 Japan; 2 Niigata Livestock Research Center, Niigata, 955-0143 Japan

**Keywords:** Plant ecology, Environmental monitoring

## Abstract

Case studies on the assessment of local organic fertilisers (OFs) in their quality (decomposition characteristics and nutrient availability for crops) are few in sub-Saharan Africa (SSA). This study assessed the quality of local OFs from the Ethiopian Rift Valley. The decomposition characteristics were assessed by acid detergent fibre analysis methods, while the short-term nitrogen availability was assessed by a combination of laboratory incubations and inorganic nitrogen and acid detergent soluble nitrogen determinations. A commercial hand-held reflectometer (RQFlex) was used for determining nitrogen components. The mean acid detergent soluble organic matter contents exceeded 250 mg g^−1^, indicating the OF feedstock contained much of the readily decomposable organic matter. Some of the indigenous compost (*kosi*) samples showed net nitrogen immobilisation during the initial period of incubation, followed by net nitrogen mineralisation in one month of incubation with 4% of the nitrogen mineralisation rate. *Kosi* should be applied to a field one month before seeding. Short-term nitrogen availability from *kosi* was too low to substitute for inorganic fertilisers. The combination of the simple analysis methods shown in this study is a quick, cost-saving, and accurate quality assessment approach for OFs, which can be useful in the field and at experimental stations in SSA.

## Introduction

In broader parts of Eastern and Southern Africa, cattle are the most important livestock and are kept for ploughing, milk, and manure uses^1^. These areas also suffer soil fertility decline due to high agricultural and grazing pressures^2^. To overcome low soil fertility problems, most of the farmers are constrained by a shortage of cash to use inorganic fertilisers (IFs); however, farm-level fertiliser prices in sub-Saharan Africa (SSA) are among the highest in the world^3^. Many medium-term (over 5 years and more) soil fertility management experiments conducted in SSA invariably showed that the best results in terms of medium-term sustained yield response were from those treatments that combined inorganic and organic inputs^4^. Given these contexts, the combined use of organic fertilisers (OFs) and IFs has been advocated in SSA since the 1990s^5^.

The major limitation in the effectiveness of OFs in SSA involves their quality and quantity; first, farmers cite quantity as a problem, that OFs are usually not enough, and second, the quality of OFs concerning nutrient release and crop uptake is poor^6^. Commonly available organic resources among smallholder farmers in SSA have divergent qualities^7^, which decompose and release various qualities of nutrients in soils at different times^8^, reflecting differences in nutrient availability and overall crop yields from each resource^9^. Besides these two parameters, i.e., decomposition characteristics and nutrient availability, maturity and stability are other important parameters for quality assessment of OFs^10^. A well-accepted definition of compost stability is the rate or degree of organic matter (OM) decomposition. Compost maturity generally refers to the degree of decomposition of phytotoxic organic substances produced during the active composting phase and to the absence of pathogens and visible weed seeds^11^.

Case studies on the quality assessment of local OFs in SSA include: (i) analyses of different decomposition characteristics and nutrient availability to establish a decision-support system for farmers^8,12^; (ii) analyses of how farmers’ management practices affect chemical composition of manure^13–15^; (iii) analysis of the relationship between farmers’ simple methods for manure/compost quality assessment and those chemical compositions^16^; and (iv) analyses of how different production and storage methods of cattle manure affected the manure organic N composition and release in soil^17^. Of these previous studies, (i) discussed decomposition characteristics of OFs and (iv) discussed N availability from OFs for crops.

A challenge is to determine how researchers can apply the enhanced predictive understanding of the two quality parameters of OFs, decomposition characteristics and nutrient availability, to assist farmers in managing their organic resources^18^. To address this, first, a minimum set of organic resource quality parameters (macronutrients, total carbon (C), lignin, soluble C, soluble polyphenolics, α-cellulose, and ash) was recommended^12^, and the resource quality of a variety of crop residues, agroforestry trees and shrubs, and leguminous cover crops were compiled into an Organic Resource Database^19^ in Eastern and Southern Africa.

Second, a decision-support system, which makes practical recommendations for appropriate use of organic materials, based on their total nitrogen (N), lignin, and polyphenol contents, was developed^19^. Gachengo *et al*. (ref.^8^) assessed the nutrient supply characteristics of 32 OF and organic materials (a standard sample set) commonly used for soil fertility management in Kenya by a proximate analysis that used the resource quality parameters of total N, lignin, and polyphenol contents and categorised them into the four quality categories (Classes I–IV). Simultaneously, this standard sample set was analysed by 28-day aerobic incubations^20^, *in vitro* dry matter digestibility^21^, and near-infrared reflectance spectrometry^22^. Regression analyses showed that the incubation analysis and *in vitro* digestibility were still best explained by the three quality parameters, N, lignin, and soluble polyphenol contents, which had been obtained from the proximate analysis^23^. When considerations of cost and speed are included in the analysis, aerobic incubation is one of the cheapest, but it’s also the slowest method. Near-infrared reflectance spectrometry is the fastest method, but also the most expensive^23^.

In the decision-support system, cattle manure was categorised into Class II that was characterised by >2.5%N^8^. A literature review showed that total N contents of the cattle manure/compost samples collected from *kraals* (cattle parking lots) and cattle sheds in local farmers’ backyards in Eastern and Southern Africa ranged from 1.1% to 1.7% with a mean of 1.2% (Table 1). Fast compost samples reviewed from two papers contained 0.7%N and 2.0%N (Table 1). It can be difficult to strictly identify the type of OFs in the field, particularly manure or compost, in the situation that “farmers reported adding organic materials directly to the manure heap, organic materials of which were rejected fodder, maize stover (used by 87% of the farmers), banana residues (71%), Napier grass (47%), roadside grass (42%), *Grevillea* leaves (34%), and other materials (16%)^16^”. Thus, representative cattle manure/compost samples collected from farmers’ backyard contained less than 2.5%N, rather classified into Classes III or IV (both categories were characterised by <2.5%N) in the decision-support system.

**Table 1:** Chemical contents of manure and compost samples collected from *kraals* and animal sheds in local farmers’ backyards in Eastern and Southern Africa.

Manure and compost	*n*	N (%)			P (%)			K (%)			C:N		
		mean	min.	max.	mean	min.	max.	mean	min.	max.	mean	min.	max.
Cattle manure (compost)^7,9,13,14,16,29,55–57^	506	1.2	1.1	1.7	0.3	0.2	0.6	2.1	0.8	2.4	23	12	29
Sheep and goat manures^7,56^	75	1.5	1.5	1.5	0.2	0.2	0.4	3.0	1.4	3.3	22	22	22
Chicken manure^7,55,56^	87	3.2	2.4	3.8	0.4	0.4	1.6	2.2	0.9	2.4	10	9	17
Fast compost^7,29^	83	1.4	0.7	2.0	0.3	0.2	0.4	1.8	1.4	1.8	13	10	18

Thus, case studies on the quality assessment of local OFs, i.e., their decomposition characteristics and nutrient availability for crops, are few in SSA. The first objective of this study is to assess decomposition characteristics of local OFs and their feedstock and to estimate a short-term N availability from those OFs, which can substitute for IFs, in the semi-arid northern Ethiopian Rift Valley. The second objective is to show an example of cheap, fast, and yet accurate analysis methods for the quality assessment of local OFs in the field and at agricultural experiment stations near the field.

Decomposition characteristics of the OFs and feedstock were assessed using acid detergent fibre methods. The short-term N availability from the OFs was estimated by laboratory incubations and inorganic N and acid detergent soluble nitrogen (ADSN)^24^ determinations.

## Materials and Methods

### Study area

Most of the Tebo and Geldia seasonal rivers catchments (the study area) are located in the semi-arid northern Ethiopian Rift Valley. The catchment areas are categorised into the two sub-areas in terms of major maize growing areas in Ethiopia (these two categories cover 63% of the total maize growing area in Ethiopia): mid-altitude dry (1000–1600 m a.s.l.; annual rainfall 800–1000 mm) and mid-altitude moist (1600–1800 m a.s.l.; annual rainfall 1000–1250 mm) sub-areas. Major crops in the mid-altitude dry sub-area of the catchments are sorghum, tef (*Eragrostis tef*), and maize, whereas those in the mid-altitude moist sub-area are wheat, tef, and maize.

Most households in the semi-arid northern Ethiopian Rift Valley hold continuously cropped maize fields (locally referred to as *aradas*), which acquire fertility from the regular input of OFs, such as compost (locally, *kosi*) or household wastes^25^. *Kosi* is made from a variety of locally available organic materials, such as various types of animal dung, kitchen ash, crop residues, and feed refusals. These compost materials are piled up in the corners of house-yards for several months to a few years for decomposition^25^. It is mainly a housewife who collects these organic materials through house-yard sweeping and dumps on a *kosi* pile. In most cases, farmers carry *kosi* from *kosi* piles to a field from May to June before crop seeding and scatter it on the ground. The *kosi* is incorporated into the soil by subsequent ploughings. Household wastes are substantially a variety of organic materials themselves that also comprise *kosi*, which is collected through house-yard sweeping and is dumped directly on an *arada* every few days.

Chemical composition analyses of *kosi* organic feedstock (Table 2) showed the animal dungs tested had higher nutritional values than secondary organic materials, such as crop residues and feed refusals, with minor exceptions across the chemical composition analysed (Table 2). Although these secondary organic materials were added, the N content of the *kosi* was comparable to that of sheep and goat manures in Eastern and Southern Africa (Tables 1 and 2). This can be because, although the primary organic material that constituted the *kosi* was cattle dung, the *kosi* also contained chicken, sheep, goat, and donkey dungs, which had higher N contents than cattle dung (Table 2). All cattle, sheep, donkeys, and chicken are parked in the same backyard at night^26^. Probably because of the higher N content, the mean C:N ratio of the *kosi*, 18, is lower than that of cattle manure/compost in Eastern and Southern Africa, 23 (Tables 1 and 2); however, either value is higher than a standard of stable compost, 10–15^27^. The *kosi* had slightly higher phosphorus (P) contents than the standard of cattle manure/compost in Eastern and Southern Africa (Tables 1 and 2), probably because of kitchen ash that was dumped on the *kosi* pile (Table 2).

**Table 2:** Nutrient contents of organic fertilisers and organic materials from the study area (mean ± SD). T-tests were conducted between the *kosi* and fast compost.

Items	*n*	N (%)	P (%)	K (%)	TOC (%)	C:N	Ca (%)	Mg (%)	Na (%)
*Kosi*	28	1.52** ± 0.56	0.35* ± 0.18	1.33^***ns***^ ± 0.59	22.49** ± 12.69	18^***ns***^ ± 8	2.98^***ns***^ ± 1.11	0.55^***ns***^ ± 0.33	0.19^***ns***^ ± 0.08
Fast compost	10	0.89** ± 0.23	0.24* ± 0.08	1.19^***ns***^ ± 0.71	11.05** ± 4.38	19^***ns***^ ± 4	2.36^***ns***^ ± 0.92	0.42^***ns***^ ± 0.29	0.19^***ns***^ ± 0.05
Maize stover	6	1.08 ± 0.28	0.11 ± 0.06	2.73 ± 0.07	49.88 ± 1.01	48 ± 10	0.43 ± 0.06	0.10 ± 0.02	0.21 ± 0.02
Sorghum stover	6	0.71 ± 0.17	0.12 ± 0.02	1.70 ± 0.13	49.95 ± 1.65	75 ± 22	0.94 ± 0.09	0.61 ± 0.25	0.05 ± 0.02
Tef straw	6	1.10 ± 0.18	0.15 ± 0.04	1.69 ± 0.09	50.27 ± 0.68	47 ± 7	1.34 ± 0.40	0.08 ± 0.04	0.11 ± 0.01
Feed refusals	6	1.47 ± 0.28	0.19 ± 0.02	1.93 ± 0.34	35.45 ± 6.40	23 ± 4	3.79 ± 1.05	0.25 ± 0.04	0.36 ± 0.13
Kitchen ash	6	0.54 ± 0.16	0.64 ± 0.15	4.39 ± 1.67	2.32 ± 2.14	5 ± 5	10.90 ± 5.16	0.71 ± 0.25	0.36 ± 0.13
Cattle dung	6	1.64 ± 0.12	0.25 ± 0.02	1.87 ± 0.60	36.76 ± 7.90	24 ± 2	2.83 ± 0.32	0.26 ± 0.13	0.28 ± 0.18
Sheep/goat dung	6	2.48 ± 0.41	0.26 ± 0.05	3.23 ± 0.62	34.81 ± 4.26	15 ± 2	4.06 ± 0.28	0.40 ± 0.05	0.47 ± 0.05
Donkey dung	6	1.95 ± 0.36	0.36 ± 0.05	2.31 ± 0.74	35.81 ± 4.10	18 ± 4	3.81 ± 1.62	0.39 ± 0.11	0.32 ± 0.02

**P < 0.01, *P < 0.05, *ns* not significant. Dry matter content and chemical contents of N (the wet Kjeldahl method), P (the vanadomolybdophosphoric acid method), K, Ca, Mg, and Na (the atomic absorption spectroscopy method)^58^, and organic C (Walkley-Black dichromate method)^59^ were analysed at the Soil and Water Analysis Laboratory of Horticoop Ethiopia PLC (Debre Zeit) in 2015. A wet triacid (HNO_3_/HClO_4_/H_2_SO_4_) digestion procedure was used for P and K. Organic C contents determined were recalculated into those determined by dry combustion methods using autoanalysers^60^.

It has been since the beginning of the 2000s when the district agriculture office began giving fast compost training to farmers^28^. The fast compost is made from the same variety of organic materials as *kosi* and household wastes. The fast compost had significantly lower N (*P* < 0.01) and P (*P* < 0.05) than the *kosi* (Table 2). Both the total N content and C:N ratio of the fast compost were the same level as those of the fast compost made by the farmers after training in Ethiopia (N = 0.7, C:N ratio = 18)^29^. Nutrient contents of the fast compost had another feature of low standard deviations across the chemical composition analysed (Table 2). The farmers attending the fast compost training were instructed on the varieties of organic materials and how to pile them up. The lower standard deviation values indicated that the trainees made the fast compost strictly following the instructions. On the other hand, the nutrient contents of the fast compost in Eastern and Southern Africa had the highest variations among the OFs across the chemical composition analysed (Table 1); e.g., the lowest N content was 0.7^29^, while the highest was 2.0^7^. This suggests that the varieties and volumes of organic materials used for making fast compost differ widely from one another.

### Sample collection

For assessing decomposition characteristics of OFs and organic materials, *kosi* (*n* = 6) and representative organic materials of *kosi* (maize and sorghum stovers, tef straw, feed refusals, kitchen ash, dried cattle dung, dried goat/sheep dung, and dried donkey dung; *n* = 3 for each) were sampled from farmers’ backyards in each of the mid-altitude dry and mid-altitude moist sub-areas. Five farmers who had participated in the fast compost training from each sub-area were requested to make compost, from which the fast compost samples were collected; i.e., 12 *kosi*, 10 fast compost, and 6 samples for each organic material in total. Similarly, for predicting short-term N availability from OFs and organic materials, 5 *kosi*, 5 fast compost, and 5 household wastes samples were collected from each of the sub-areas; i.e., 10 *kosi*, 10 fast compost, and 10 household wastes samples in total.

For making the fast compost samples, organic materials collected from the farmers’ backyards were piled up in a rectangular wooden frame (1 m in width and length and 1.5 m in depth). Following the technical guidance of Ministry of Agriculture and Rural Development (ref.^28^), each 20-cm-deep layer of the (i) maize and sorghum stalks, (ii) animal dungs, and (iii) tef residue and feed refusals were piled up in turn until it reached the top of the pile. The total depth of each material layer was arranged to be the same between (i), (ii), and (iii). Kitchen ash (0.5 kg m^−2^) was sprinkled over each layer of the (i) and (iii). Some humic soil (1–2 cm deep) was spread on top of each layer. Water was regularly added to keep the pile moist. Once every 21 days, all the organic materials in the pile were turned over to mix the materials. This process was repeated to make compost ready in 3 months^28^.

### Decomposition characteristics

**Figure 1:**
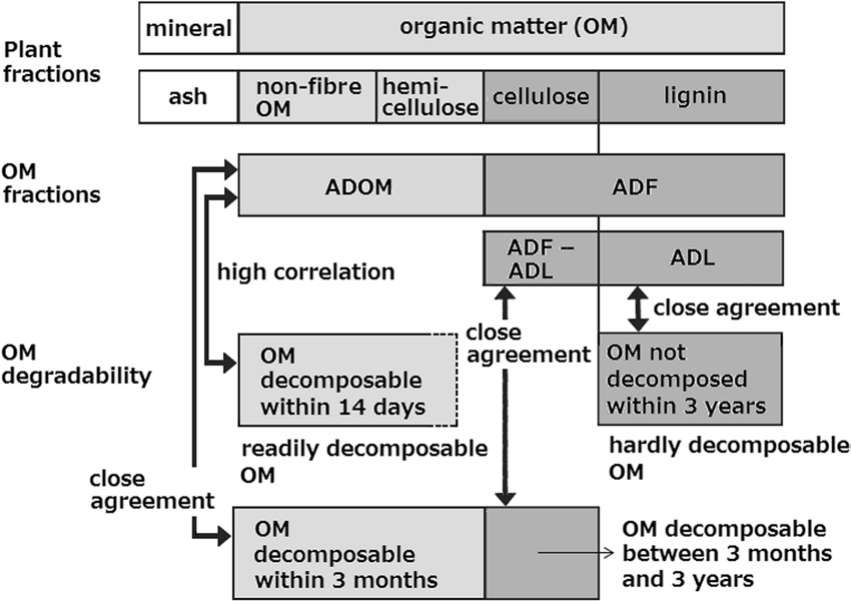
Relationship between plant fractions, organic matter fractions, and organic matter degradability. OM, organic matter; ADOM, acid detergent soluble organic matter; ADF, acid detergent fibre; ADL, acid detergent fibre lignin. Fig. 2 in Oyanagi *et al*. (ref.^32^) and Fig. 2 in Oyanagi *et al*. (ref.^35^) were integrated.

Acid detergent fibre methods have recently been used for a proximate analysis to assess decomposition characteristics of OFs and organic materials^30–34^. By burying OFs and organic materials underground for 3 months^32^ and 3 years^35^ using the glass fibre-filter paper bag methods^33^ and conducting laboratory incubations (30 °C, 14 days)^36^, Oyanagi *et al*. (refs^32,35,36^) confirmed that acid detergent soluble OM (ADOM; ADOM represents OM other than acid detergent fibre, ADF, that is the sum of non-fibre OM and hemi-cellulose; Fig. 1), ADF, and acid detergent fibre lignin (ADL) can be used for indices to characterize readily decomposable OM (OM decomposable within 14 days), OM not decomposed within 3 months, and hardly decomposable OM (OM not decomposed within 3 years), respectively, for OFs and organic materials in general (Fig. 1; see Decomposition characteristics and Figs. S1, S2 and S3 in Supplementary Information).

In this study, for the *kosi*, fast compost, and organic materials, OM degradability (Fig. 1), i.e., readily decomposable OM represented by the equation of 0.365 ADOM – 56^36^ (see Fig. S2 in Supplementary Information), OM decomposable within 3 months represented by ADOM (i.e., 1000–ADF–crude ash; see Fig. S1), and OM decomposable within 3 years represented by 1000–ADL–crude ash (see Fig. S3), were assessed using the acid detergent fibre analysis methods^37,38^.

N components were determined using a commercial hand-held reflectometer (Merck RQFlex). RQFlex has recently proved to be a rapid on-site estimator of total N (R^2^ = 0.95 with the wet Kjeldahl method, *n* = 98^34^), NH_4_^+^ (R^2^ = 0.91 with the stream distillation, *n* = 107^39^; R^2^ = 0.96 with the stream distillation, *n* = 36^40^), and NO_3_^−^ (R^2^ = 0.995 with the stream distillation, *n* = 98^34^) in manure and organic materials. In this study, total N determination followed the methods shown in Ando *et al*. (ref.^34^; see Simple determination method of total N using RQFlex in Supplementary Information).

Based on the values of crude ash (representing mineral), OM, 0.365 ADOM – 56 (similarly, readily decomposable OM representing OM decomposable within 14 days), ADOM (OM decomposable within 3 months), ADF – ADL (OM decomposable between 3 months and 3 years), and 1000 – ADL – ash (OM decomposable within 3 years) obtained, (i) an analysis of variance was conducted to detect differences in these mean values between each pair of the organic materials and (ii) a cluster analysis and canonical discriminant analysis were conducted to get cluster numbers and discriminant scores of the each organic material sample, based on which all the samples were grouped. SPSS ver. 20 (IBM) was used for the statistical analyses.

Meanwhile, the *kosi* sample contained soil, which was mixed in the process of house-yard sweeping, while the fast compost sample contained humic soil. These made determinations of a crude ash content and OM fractions (ADOM, ADF, and ADL) difficult (particularly, ADOM is affected by the crude ash content in the calculation process). However, it is possible to compare the ratios between some indicators of the OM degradability to assess decomposition characteristics of the *kosi* and fast compost. Kruskal-Wallis tests were applied to detect differences (OM decomposable within 14 days)/(OM decomposable between 3 months and 3 years), (OM decomposable within 14 days)/(OM decomposable within 3 years), (OM decomposable within 3 months)/(OM decomposable between 3 months and 3 years), (OM decomposable within 3 months)/(OM decomposable within 3 years), and (OM decomposable between 3 months and 3 years)/(OM decomposable within 3 years) between each pair of the OFs and organic materials.

### Laboratory incubation for predicting short-term N availability

Laboratory incubations were conducted at room temperature (20–30 °C) and 60% saturation with water for 42 days. An OF sample containing 30 mgN was mixed with 100 g of the soil collected from an *arada* field (*Endopetric Hypercalcic Calcisol*; clay loam), which was placed in a 100-mL polyethylene bottle. In 3, 7, 14, 21, 28, 35, and 42 days of incubation, 100 mL of 0.5 mol L^−1^ hydrochloric acid was added to 10 g of the sample and stirred by a mixer for two minutes to make an extract^41^ (see Hydrochloric acid extraction method to determine NH_4_^+^ and Fig. S4 in Supplementary Information). The extract was diluted, if necessary, and was reacted with Reflectquant ammonium test (0.2–7.0 mg L^−1^ NH_4_^+^) and Reflectquant nitrate test (5–225 mg L^−1^ NO_3_^−^) to determine NH_4_^+^ and NO_3_^−^ contents with an RQFlex, respectively. Net N mineralised was calculated by subtracting NH_4_^+^ and NO_3_^−^ concentrations of an incubated control (the soil without OF) from NH_4_^+^ and NO_3_^−^ concentrations of an incubated sample.

Stability/maturity indices established for composts of various origins, <0.4 mg g^−1^ for NH_4_^+^ ^42^, >0.3 mg g^−1^ for NO_3_^−^ ^43^, and <1 for NH_4_^+^:NO_3_^−^ ratio^44^ were used for assessing stability/maturity of the three OFs.

### Inorganic N and ADSN determinations for predicting short-term N availability

Acid detergent fibre analysis methods have recently been used for a proximate analysis to predict available N from OFs^24,45,46^. Based on the results of laboratory incubations (30 °C, 84 days) for different types of composted manures^21,46^ (see Inorganic N and ADSN determinations for predicting short-term N availability and Figs. S5 and S6 in Supplementary Information), Oyanagi *et al*. (ref.^47^) concluded that N available from an OF that contains <250 mg g^−1^ ADOM (95% of the composted cattle manures used in the laboratory incubations^46^ belonged to this category) in the first application year can be estimated only by inorganic N in the OF. For an OF that contains ≥250 mg g^−1^ ADOM, 4-week (approximately 1 month) N availability can be assessed only by inorganic N in the OF, while 12-week (approximately 3 months) N availability can be assessed by the equation of 0.50 ADSN – 2.5 (ADSN is N in ADOM).

The *kosi* and fast compost contained soil, which made a determination of ADOM difficult. In this study, therefore, 12-week N availability was assessed by the equation of 0.50 ADSN – 2.5, while 4-week N availability was assessed by inorganic N in the OF. N mineralisation rates, i.e., the proportions of N available in total N of the OF, were calculated in 4 weeks and 12 weeks of incubation to obtain 4-week and 12-week N mineralisation rates, respectively.

Multiple comparison tests (the Bonferroni correction) were conducted to detect differences in the mean values of inorganic N concentrations (NH_4_^+^ and NO_3_^−^), the ratio of NH_4_^+^ to NO_3_^−^ (NH_4_^+^:NO_3_^−^), 4-week and 12-week N availability, and 4-week and 12-week N mineralisation rates between each pair of the OF samples (*kosi*, fast compost, and household wastes).

## Results

### Decomposition characteristics

**Figure 2:**
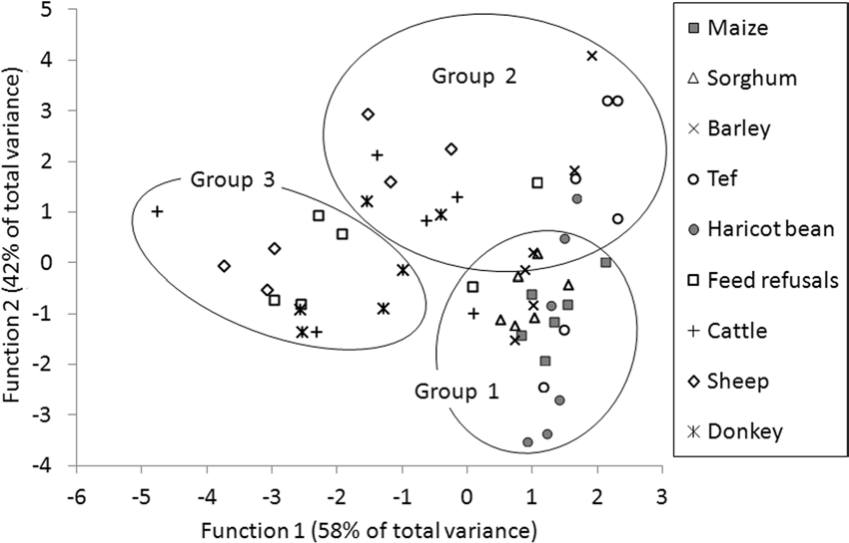
Scatter graph of 1^st^ canonical discriminant score and 2^nd^ canonical discriminant score. Groups 1 to 3 represent the cluster analysis.

The cluster analysis showed the organic material samples were classified into three groups (Fig. 2). The discrimination analysis showed the 1^st^ and 2^nd^ standardized canonical discriminant functions accounted for 58% and 42% of the total variance, respectively, being 99.8% of the total variance in total. Thus, the 1^st^ and 2^nd^ canonical scores were used to visualise how well the discriminant functions classified the data.

Both the groups 1 and 2 were mainly composed of the samples of crop residues. Both groups commonly had a higher OM (i.e., lower mineral) and the higher OM decomposable between 3 months and 3 years (i.e., higher cellulose or ADF–ADL; Table 3). The samples of maize and sorghum stovers and haricot beans shell and stalk were mainly of group 1, while the samples of barley and tef straws were mainly of group 2. Group 2 was also characterised by the rich OM decomposable within 3 months (i.e., ADOM) including the OM decomposable within 14 days (i.e., readily decomposable OM). Both the OM decomposable within 14 days and the OM decomposable within 3 months of the barley and tef straws was the highest among the organic materials, although no significant difference was observed between the organic materials.

**Table 3:** Decomposition characteristics of organic fertilisers and organic materials from the study area (mean ± SD, dry matter).

Items	*n*	Groups^a^	Mineral and OM contents and indicators of OM degradability (mg g^−1^)^b^						Ratios between some indicators of OM degradability^c^				
			Mineral	OM	(a) within 14 days	(b) within 3 months	(c) 3 mos.–3 yrs.	(d) within 3 years	(a)/(c)	(a)/(d)	(b)/(c)	(b)/(d)	(c)/(d)
Maize stover	6	1	106 ± 17^**a**^	894 ± 17^**a**^	60 ± 20^***ns***^	318 ± 54^***ns***^	434 ± 54^**a**^	751 ± 71^**ac**^	0.1 ± 0.1^**a**^	0.1 ± 0.0^**a**^	0.8 ± 0.1^**ab**^	0.4 ± 0.0^**ab**^	0.6 ± 0.0^**ab**^
Sorghum stover	6	1, 2	109 ± 22^**a**^	891 ± 22^**a**^	60 ± 15^***ns***^	318 ± 42^***ns***^	354 ± 40^**ac**^	672 ± 44^**ab**^	0.2 ± 0.0^**b**^	0.1 ± 0.0^**a**^	0.9 ± 0.2^**ab**^	0.5 ± 0.0^**ab**^	0.5 ± 0.0^**ab**^
Haricot beans	6	1, 2	131 ± 26^**a**^	869 ± 26^**a**^	45 ± 51^***ns***^	243 ± 182^***ns***^	505 ± 157^**ab**^	747 ± 33^**ac**^	0.3 ± 0.3^**b**^	0.1 ± 0.1^**a**^	0.6 ± 0.6^**a**^	0.3 ± 0.2^**a**^	0.7 ± 0.2^**a**^
Barley straw	6	1, 2	126 ± 19^**a**^	874 ± 19^**a**^	91 ± 50^***ns***^	403 ± 137^***ns***^	315 ± 101^**ab**^	719 ± 47^**acd**^	0.3 ± 0.4^**b**^	0.1 ± 0.1^**a**^	1.6 ± 1.5^**ab**^	0.6 ± 0.2^**ab**^	0.4 ± 0.2^**ab**^
Tef straw	6	1, 2	96 ± 14^**a**^	904 ± 14^**a**^	106 ± 55^***ns***^	445 ± 150^***ns***^	345 ± 153^**ab**^	790 ± 48^**c**^	0.3 ± 0.3^**b**^	0.1 ± 0.1^**a**^	1.8 ± 1.3^**ab**^	0.6 ± 0.2^**ab**^	0.4 ± 0.2^**ab**^
Feed refusals	6	1, 2, 3	335 ± 126^**ab**^	665 ± 126^**ab**^	45 ± 42^***ns***^	256 ± 142^***ns***^	284 ± 59^**bc**^	539 ± 126^**ac**^	0.1 ± 0.2^**ac**^	0.1 ± 0.1^**ac**^	1.0 ± 0.6^**ab**^	0.4 ± 0.2^**ab**^	0.6 ± 0.2^**ab**^
Cattle dung	6	1, 2, 3	352 ± 154^**ab**^	648 ± 154^**ab**^	49 ± 42^***ns***^	291 ± 156^***ns***^	271 ± 111^**ab**^	535 ± 179^**ac**^	0.2 ± 0.2^**ac**^	0.1 ± 0.1^**ac**^	1.3 ± 1.1^**ab**^	0.5 ± 0.3^**ab**^	0.5 ± 0.3^**ab**^
Sheep/goat dung	6	2, 3	382 ± 86^**b**^	618 ± 86^**b**^	56 ± 49^***ns***^	295 ± 152^***ns***^	180 ± 53^**b**^	475 ± 128^**b**^	0.2 ± 0.2^**ac**^	0.2 ± 0.2^**a**^	1.9 ± 1.4^**b**^	0.6 ± 0.2^**b**^	0.4 ± 0.2^**b**^
Donkey dung	6	2, 3	327 ± 57^**b**^	673 ± 57^**b**^	39 ± 32^***ns***^	259 ± 88^***ns***^	286 ± 50^**bc**^	545 ± 61^**bd**^	0.4 ± 0.4^**a**^	0.1 ± 0.1^**a**^	1.0 ± 0.5^**ab**^	0.5 ± 0.1^**ab**^	0.5 ± 0.1^**ab**^
*Kosi*	12		674 ± 143	326 ± 143	10 ± 19	125 ± 94	119 ± 49	247 ± 130	0.1 ± 0.1^**ac**^	0.0 ± 0.0^**bc**^	1.0 ± 0.5^**ab**^	0.5 ± 0.1^**ab**^	0.5 ± 0.1^**ab**^
Fast compost	10		674 ± 23	326 ± 23	3 ± 4	139 ± 44	120 ± 38	259 ± 44	0.0 ± 0.1^**c**^	0.0 ± 0.0^**b**^	1.2 ± 0.7^**ab**^	0.5 ± 0.1^**ab**^	0.5 ± 0.1^**ab**^

^a^Cluster analysis and canonical discriminant analysis were conducted to get cluster numbers of each organic material sample. ^b^Analysis of variance was conducted between each pair of the organic materials. Different superscript letters between the organic materials indicate statistically significant differences (*P* < 0.05). *ns* not significant. ^c^Kruskal-Wallis tests were applied between each pair of the organic fertilisers and organic materials. OM, organic matter; a) OM fraction that is decomposable within 14 days, i.e., readily decomposable OM; b) OM fraction that is decomposable within 3 months, i.e., ADOM (acid detergent soluble organic matter); c) OM fraction that is decomposable between 3 months and 3 years, i.e., ADF (acid detergent fibre)–ADL (acid detergent fibre lignin); d) OM fraction that is decomposable within 3 years, i,e., 1000–ADL–ash.

Group 3 was mainly composed of the livestock dungs, which had the highest proportions of mineral (crude ash), and every indicator that represents OM degradability of group 3 was the lowest among the organic materials (except for the haricot beans shell and stalk; Table 3). The feed refusals spanned all the groups, probably because it was a mixture of livestock dungs and feeds, i.e., crop residues.

The *kosi* and fast compost had higher mineral contents, partly because it was likely to be affected by the soil contained (Table 3). Between the *kosi* and fast compost, no noticeable difference was observed in the indicators that represent OM degradability and the ratios between some indicators of the OM degradability. Of the 12 *kosi* and 10 fast compost samples, 8 and 4 samples contained substantially no OM fraction that is decomposable within 14 days, respectably, and the two OFs probably contained <250 mg g^−1^ ADOM. The (OM decomposable within 14 days)/(OM decomposable between 3 months and 3 years) of the two OFs were significantly lower than that of the most crop residues. The (OM decomposable within 14 days)/(OM decomposable within 3 years) of the *kosi* was significantly lower than that of all the crop residues, sheep/goat dung, and donkey dung, and that of the fast compost was significantly lower than that of all the organic materials. These suggest that the OM fraction that was decomposable within 14 days was more rapidly decomposed than other fractions of OM degradability in the composting process of the two OFs.

### Short-term N availability

The multiple comparison tests showed that NH_4_^+^ in the *kosi* and fast compost were significantly lower than that in the household wastes (Table 4). NO_3_^−^ in the three OFs significantly differed from one another, being higher in the order of the *kosi*, fast compost, and household wastes. Only the *kosi* satisfied all the stability/maturity indices, whereas NO_3_^−^ in the fast compost was lower than the critical limit value of stable/mature compost. The household wastes did not satisfy the stability/maturity indices of either NO_3_^−^ or NH_4_^+^:NO_3_^−^ ratio, probably still being in the composting process.

**Table 4:** NH_4_^+^ and NO_3_^−^ concentrations, NH_4_^+^:NO_3_^−^ ratios, 4-week and 12-week N availability, and 4-week and 12-week N mineralisation rates for the *kosi*, fast compost, and household wastes (mean ± SD).

Organic fertilisers	*n*	NH_4_^+^ (mg g^−1^)	NO_3_^−^ (mg g^−1^)	NH_4_^+^:NO_3_^−^	N availability (mg g^−1^)		Mineralisation rates (%)	
					4-week	12-week	4-week	12-week
*Kosi*	10	0.011 ± 0.006^a^	0.50 ± 0.29^a^	0.03 ± 0.02^a^	0.51 ± 0.29^a^	1.37 ± 1.08^a^	4.0 ± 1.6^a^	11.2 ± 8.9^a^
Fast compost	10	0.007 ± 0.001^a^	0.23 ± 0.07^b^	0.03 ± 0.01^a^	0.23 ± 0.07^b^	0.23 ± 0.07^b^	3.9 ± 1.2^a^	3.9 ± 1.2^a^
Household wastes	10	0.085 ± 0.055^b^	0.04 ± 0.07^c^	7.58 ± 6.57^b^	0.13 ± 0.06^c^	2.73 ± 0.94^c^	2.3 ± 1.7^a^	54.0 ± 25.8^b^

Different superscript letters between the organic fertilisers indicate statistically significant differences (*P* < 0.05). *ns* not significant.

Inorganic N and ADSN determinations predicted that 4-week N availability from *kosi* will be significantly higher than that from fast compost and household wastes (Table 4). Additionally, because higher amounts of organic N will be mineralised from *kosi* and household wastes during the subsequent 8 weeks than from fast compost, 12-week N availability will be higher in the order of household wastes, *kosi*, and fast compost. While approximately 50% of total N will be mineralised in 12 weeks after a household wastes application, 12-week N mineralisation rates will be approximately 11% and 4% for *kosi* and fast compost, respectively.

**Figure 3:**
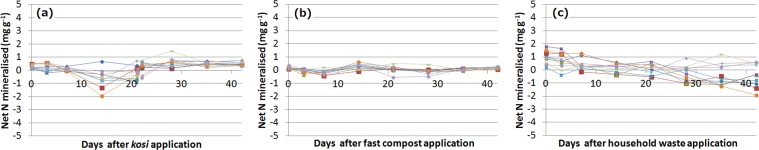
Net N mineralised during the 42-day incubation for the *kosi* (**a**), fast compost (**b**), and household wastes (**c**) samples (*n* = 10 for each). Different colour lines in each graph showed different samples.

Until 28 days of incubation, some of the *kosi* samples showed net N immobilisation, whereas the fast compost samples showed no noticeable change in net N mineralised (Fig. 3). In 28 days of incubation, all the net N mineralised of the *kosi* and fast compost exceeded 0 mg g^−1^, and net N mineralised in 35 and 42 days of incubation for the *kosi* were on average 0.48 and 0.49 mg g^−1^, respectively, and those of the fast compost were 0.08 and 0.19 mg g^−1^. All the household wastes samples had shown a slight N immobilisation until 21 to 28 days of incubation. Since then, they were divided into two groups: one N mineralisation of which prevailed (4 samples); another N immobilisation of which prevailed (6 samples).

## Discussion

The mean net N mineralised from the *kosi* and fast compost in 42 days of incubation (Fig. 3) were almost equal to inorganic N in the *kosi* and fast compost, i.e., 4-week N availability (Table 4), respectively. This suggests that mineralised N that was derived from easily decomposable OM in approximately 1 month of incubation was nearly 0, which was also supported by the OM fraction that is decomposable within 14 days (readily decomposable OM) contained in the *kosi* and fast compost (Table 3). The fast compost mean 12-week N availability was the same as the 4-week value (Table 4), indicating N mineralised from easily decomposable OM will continuously be negligible even approximately 3 months after a fast compost application. The amount of N available from manure includes the inorganic content of manure (NH_4_^+^ and NO_3_^−^) plus the amount of organic N mineralised following application^48^. Both NH_4_^+^ and NO_3_^−^ in the fast compost were lower than those in the *kosi*, and the amount of organic N mineralised following the fast compost application will be 0 because the fast compost probably contained <250 mg g^−1^ ADOM.

This is in line with the prediction of short-term N availability from most of the cattle manures/composts shown by Tanahashi & Oyanagi (ref.^46^; see Inorganic N and ADSN determinations for predicting short-term N availability and Fig. S5 in Supplementary Information). Markewich *et al*. (ref.^17^) conducted a litterbag experiment in Kenya with cattle manure samples with different feed qualities and storage methods and found that more of the refractory fibre-bound N remained in the low-quality manure, not mineralised during 4 months in soil. Eghball *et al*. (ref.^48^) found that organic N mineralized during the corn growing season (5 months) from composted cattle manure was about half (11%) of that for noncomposted cattle feedlot manure (21%). Eghball *et al*. (ref.^48^) noted that lower N mineralisation from compost was because most of the easily convertible C and N compounds were lost during the composting process and the remaining C and N were in more stable forms. The rapid decomposition in the OM fraction that is decomposable within 14 days found in this study (Table 3) was in line with these findings. In contrast, additional N will be mineralised from the easily decomposable OM fraction of *kosi* during the period from 4-week to 12-week. This can be because a *kosi* pile includes immature *kosi* ADOM of which exceeds 250 mg g^−1^ (ADOM of haricot beans shell and stalk was a little less than 250 mg g^−1^; Table 3) as farmers pile up a fresh organic material on a mature *kosi* pile.

In the 84-day incubation tests, Tanahashi & Oyanagi (ref.^46^) showed that ADOM was also an appropriate indicator of N mineralisation patterns. N mineralisation patterns of the composted cattle manure that had ≥250 mg g^−1^ ADOM (5% of the cattle manure samples) were characterised by an initial N immobilisation (4.7–5.3 mg g^−1^ N at maximum) occurred during the initial approximately 7 days of incubation, followed by N mineralisation (1.8–8.0 mg g^−1^ N at maximum) during the remaining incubation period. In contrast, N mineralisation patterns of the composted cattle manure that had <250 mg g^−1^ ADOM (95% of the samples) were characterised by an unclear N mineralisation pattern and minor fluctuation (0.5–1.0 mg g^−1^ of maximum N immobilisation or 1.3–1.4 mg g^−1^ of maximum N mineralisation, if any) during the entire incubation period. However, some of the composts that had 100–175 mg g^−1^ ADOM showed N immobilisations began in approximately 56 days of incubation and lasted over the remaining incubation period. Those were 4 of the 5 composted cattle manure samples that had the highest C:N ratios (26.1–42.5) of the samples. Beauchamp (ref.^49^) conducted maize yield trials with the application of IFs, chicken manure, liquid dairy cattle manure, and solid beef cattle manure for 3 years. The results showed that maize yield response to 100 kg N ha^−1^ treatments was consistently in the general order of IFs > chicken manure > liquid dairy cattle manure > solid beef cattle manure over 3 years; however, only in the 1^st^ year, maize yield with the solid manure treatment was lower than that with the control treatment (no input). This was because: (i) net N immobilisation occurred for the 1^st^ crop shortly after the solid manure (C:N ratio = 15.4) application; and (ii) lower soil NO_3_^−^ with the solid manure treatment reflected the lower availability of N. Occurrence of net N immobilisation shortly after an application of cattle manure that had a high C:N ratio (C:N ratio = 15.9) was also reported^50^. The high C:N ratio of cattle manure/compost is a feature commonly observed for *kosi* and those in Eastern and Southern Africa. The phenomena that net N immobilisation occurred initially for some of the *kosi* followed by a steady net N mineralisation to reach approximately the level of 4-week N availability during the remaining incubation period agree with the findings shown by Tanahashi & Oyanagi (ref.^46^) and Beauchamp (ref.^49^).

Farmers in Eastern and Southern Africa carry OFs from their *kraals* and cattle sheds to a field and integrate it into the field by ploughing operations carried out after a couple of weeks^51^. Temesgen *et al*. (ref.^52^) studied the purposes of tillage in the two representative areas in the semi-arid northern Ethiopian Rift Valley. Almost all the farmers (98%) in Melkawoba (relatively dry and sandy silt soil) and 50–52% of the farmers in Wulinchity (relatively humid and silty clay soil) plough maize and tef fields at planting for integrating *kosi* and household wastes into the soils. Considering the net N immobilisation possibly occurs after *kosi* application, it can be recommended to integrate the *kosi* into field soil at the ploughing time that is carried out about one month before seeding. Integration into the soil before this possibly causes loss of available N derived from *kosi*, and integration into the soil after this possibly causes a lower rate of seedling emergence and yield reduction due to N immobilisation^51^.

Immature household wastes are a mixture of fresh organic materials itself because almost all of the OF organic materials can contain much of the readily decomposable OM (OM decomposable within 14 days; ADOM ≥ 250 mg g^−1^; Table 3). Therefore, all the household wastes showed N immobilisation during 1 to 2 weeks of incubation (Fig. 3); N available to crops during this period will be quite unstable. The NH_4_^+^:NO_3_^−^ ratio of the household wastes had a great standard deviation (Table 4), ranging from the minimum value of 0.02 to the maximum value of 18.27. One household wastes sample NH_4_^+^:NO_3_^−^ ratio of which was 0.02 had 0.007 mg g^−1^ of NH_4_^+^ and 0.248 mg g^−1^ of NO_3_^−^, being comparable to those levels of the *kosi*. This indicates the household wastes samples had different maturation stages ranging from a mixture of fresh organic materials to a mixture of organic materials that had left in farmers’ backyard in a long period to pass the similar composting process to the *kosi*. Therefore, inputting a mixture of fresh organic materials directly to the field can pose more than one month of net N immobilisation after application. Farmers should stop a direct application of household wastes to the field; instead, household wastes should be piled up to make mature *kosi* and then applied to the field.

Lower short-term N availability is the other prevailing feature observed for both *kosi* and cattle manure/compost in Eastern and Southern Africa. Inorganic N in the *kosi* was comparable to that in the 141 dairy manures from Central Kenya: mean 0.49, standard deviation 0.53, minimum 0.02, and maximum 1.69 mg g^−1^ ^16^. Application of solid beef cattle manure at the rate of 100 kg N ha^−1^ in Beauchamp (ref.^49^) was equivalent to 6.6 Mg ha^−1^ of *kosi* application that contained the mean 1.52%N (Table 2). This is almost equal to the mean application rate of *kosi* to *arada*, 6.0 Mg ha^−1^ yr^−1^, in the semi-arid northern Ethiopian Rift Valley^25^. Based on the 4-week and 12-week N availability from the *kosi*, 0.51 and 1.37 mg g^−1^ (Table 4), *kosi* application to an *arada* soil with the application rate of 6.0 Mg ha^−1^ yr^−1^ can supply 3.0 and 8.2 kg N ha^−1^ yr^−1^ to the *arada*, respectively. These N availabilities are far below the recommended dose of fertiliser N use in Ethiopia, 64 kg N ha^−1^ yr^−1^ (100 kg ha^−1^ Di-Ammonium Phosphate and 100 kg ha^−1^ Urea). Given these, *kosi* and fast compost cannot be expected to contribute much to crop yield gains in the first application year.

Mukai (ref.^25^) conducted a maize yield trial in the semi-arid northern Ethiopian Rift Valley and confirmed that: (i) maize yield up to ~5.0 Mg ha^−1^ can be linearly best explained solely by the soil available N concentration; and (ii) continuous application of *kosi* at the rates of 84 kg N ha^−1^ (almost equivalent to 5.5 Mg ha^−1^ yr^−1^ application) and 164 kg N ha^−1^ (similarly, 10.8 Mg ha^−1^ yr^−1^) for over ten years increased soil available N concentrations to the 3.0 Mg ha^−1^ and 4.0 Mg ha^−1^ yield levels of maize, respectively. In the field where *kosi* has been applied for over 20 years, significant improvements in soil chemical properties in general and part of soil physical properties (decrease in soil compaction and bulk density and increase in porosity) were observed^53^. Thus, cattle manure/compost in Eastern and Southern Africa including *kosi* should not be considered to be quick-acting fertilisers; instead, it can be considered to be OFs that contribute to soil cultivation by their medium- or long-term application and to gain crop yields by the increase in total C and N in the field soil^17,54^.

Simple determination methods for N components by using RQFlex and the acid detergent fibre analysis methods used in this study can be implemented in agricultural experiment stations that equip a fume hood. These are quick, cost-saving, and yet accurate on-site quality assessment methods for OFs.

## Conclusions

The *kosi* organic materials have diversified decomposition characteristics and yet commonly contain much of the ADOM that represents the OM decomposable within 3 months. In the composting process of the *kosi* and fast compost, the readily decomposable OM fraction was more rapidly lost. The *kosi* and fast compost samples had higher C:N ratios and low inorganic N contents, which are typical characteristics to cattle manure/compost in Eastern and Southern Africa. These factors can work together, and some of the *kosi* showed initial net N immobilisation, which was followed by net N mineralisation of all the *kosi* samples and then to the level of 4-week N availability. *Kosi* should be applied to the field one month before seeding.

Many of the household wastes samples did not meet the OF stability/maturity indices, being still in the composting process. Net N immobilisation prevailed for more than half of the household wastes samples for more than one month period. Household wastes should not be applied directly to the field; instead, it should be added to a *kosi* pile and used after being mature *kosi*.

In a *kosi* application year, N availability from the *kosi* is limited, and *kosi* shows low substitutability for IFs. The present local practices of continuous *kosi* application in the mid- to long-term, are best suited.

## Supplementary information

Supplementary Information for Decomposition characteristics of indigenous organic fertilisers and introduced quick compost and their short-term nitrogen availability in the semi-arid Ethiopian Rift Valley

## Data Availability

All data analysed are included in this article (and its Supplementary Information file).
