# Supplementary material for: Decomposition characteristics of indigenous organic fertilisers and introduced quick compost and their short-term nitrogen availability in the semi-arid Ethiopian Rift Valley
Source: Sci Rep. 2019 Nov 5;9:16000. doi: 10.1038/s41598-019-52497-8 (PMC6831661; doi:10.1038/s41598-019-52497-8)
Supplement: Supplementary file 1 — Supplementary Information for Decomposition characteristics of indigenous organic fertilisers and introduced quick compost and their short-term nitrogen availability in the semi-arid Ethiopian Rift Valley [file 41598_2019_52497_MOESM1_ESM.docx]

**Supplementary Information for**

**Decomposition characteristics of indigenous organic fertilisers and introduced quick compost and their short-term nitrogen availability in the semi-arid Ethiopian Rift Valley**

Shiro Mukai & Wataru Oyanagi

**Decomposition characteristics.**

Oyanagi *et al.* (ref.**^1^**) buried 42 OFs (15 cattle, 14 swine, 6 chicken, and 7 other manures, and 7 other OFs) and 21 organic materials (3 dried raw garbage and 18 others including biological wastes) underground for 3 months using the glass fibre-filter paper bag methods**^2^** performed with 3 repetitions. For each sample, they determined C retained in 3 months of incubation in the soil (Y), acid detergent fibre fraction (ADF, X_1_), and C contained in ADF (ADF-C, X_2_). C was determined by a dry combustion method using SUMIGRAPH NC-90A, Sumika Chemical Analysis Service, Japan. They found (i) a close agreement between Y and X_2_ except for the sludge-origin samples (Fig. **S1**) and (ii) a significantly positive linear relationship between Y and X_1_ (Y = 0.581 X_1_, R^2^ = 0.885, *P* < 0.01).

Oyanagi *et al.* (ref.**^3^**) conducted laboratory incubations (30°C, 14 days) for 117 OFs (30 cattle, 43 swine, 13 chicken, and 18 other manures, and 13 other OFs) and 53 organic materials (11 raw garbage, 10 foods and food residues, 12 livestock feeds, and 20 others including biological wastes) and found a significantly positive linear regression line (Y = 0.365X – 56, *r* = 0.925, *P* < 0.01) in the relationship between C decomposed within 14 days in the soil (Y), representing readily decomposable organic matter (OM), and acid detergent soluble organic matter (ADOM; ADOM represents OM other than ADF, that is the sum of non-fibre OM and hemi-cellulose; Fig. **1**) of the sample (X) that was determined by the acid detergent fibre methods (Fig. **S2**).

Oyanagi *et al.* (ref.**^4^**) buried 18 manures (10 cattle, 6 swine, and 2 chicken) and 9 organic materials (3 livestock dung, 1 rapeseed oil cake, 1 rice bran, 1 bark compost, 1 cedar leaves, 1 muck soil, and 1 cherry tree chips) underground for 3 years using the glass fibre-filter paper bag methods performed with 3 repetitions. They found (i) a significantly positive linear relationship (Y = X_1_, R^2^ = 0.835) between C retained in 3 years of incubation in the soil (Y) and C contained in acid detergent fibre lignin fraction (ADL-C) of the sample (X_1_) (Fig. **S3**) and (ii) a significantly positive linear relationship (Y = 0.565 X_2_, R^2^ = 0.752, *P* < 0.01) between Y and ADL of the sample (X_2_) (calculated from the original data).

**Van Soest detergent fibre analysis methods used in this study.**

The sample (1 g) was mixed with 100 mL of acid detergent solution (20 g cetyltrimethylammonium bromide dissolved in 1000 mL of 0.5 mol L^-1^ sulphuric acid). The mixture was boiled for 1 hour, filtrated using a glass crucible, washed with hot water under reduced pressure, dried, and weighed (*a*). The dried residue was mixed thoroughly with 15 mL of 12 mol L^-1^ sulphuric acid and kept under room temperature for 4 hours and stirred every 30 minutes. Water was added to make its volume of 300 mL, and the mixture was boiled for 10 minutes, filtered, washed with water, dried, and weighed (*b*). The dried residue was incinerated and weighed again (*c*; silica). ADF and ADL were determined by (*a*–*c*) and (*b*–*c*), respectively. Because ADOM is OM other than ADF, it was calculated by subtracting crude ash and ADF from the dry weight of the sample. ADSN (N in ADOM) was obtained by subtracting N in (*a*) from the total N in the sample.

**Simple determination method of total N using RQFlex.**

Ando *et al.* (ref.**^7^**) digested the samples of 44 manures (14 cattle, 17 swine, 13 chicken), 11 other OFs (1 bark compost, 3 mushroom bed wastes, 7 commercial OFs), and 43 organic materials (3 food wastes, 2 active sludge, 1 dog food, 1 sawdust, 1 rice husk, 1 copy paper, 11 concentrated feeds, and 23 grass feeds) with sulphuric acid and hydrogen peroxide. The digested solution was reacted with Reflectquant ammonium tests to determine NH_4_^+^ with an RQFlex (X). They found a strong positive linear relationship (Y = 0.830 X, R^2^ = 0.949) with the determination values by the routine laboratory method, Kjeldahl wet digestion (Y).

Total N determination in this study followed the methods shown in Ando *et al.* (ref.**^7^**). After 1.0 g (dry matter) of the sample was placed in a 500-mL tall beaker, 8 mL of sulphuric acid was added and then 4 mL of 35% hydrogen peroxide was added twice, which was capped with a dish. After a vigorous chemical reaction was settled, the beaker was heated for 5 minutes. After the beaker was cooled down, 2 mL of hydrogen peroxide was added, and then 4 mL of 35% and then heated for 3 minutes; this operation was repeated 6 times. The solution was transferred to a volumetric flask and water was filled to the marked line of 100 mL. Because Reflectquant ammonium test (0.2–7.0 mg L^-1^ NH_4_^+^) requires a test solution regulated in pH 4–13, after 29 mL of water was added to 1 mL of the solution, 0.4 g of calcium hydroxide was added, which was stirred hard. The filtrate was reacted with a Reflectquant ammonium test, and NH_4_^+^ was determined with an RQFlex in a thermostat bath kept at 30°C. A standard solution for NH_4_^+^ (3.0 μg mL^-1^) was simultaneously determined to correct determined NH_4_^+^ in the sample. Corrected NH_4_^+^ in the sample (X) was converted to total N (Y) using the equation, Y = 0.830 X.

**Hydrochloric acid extraction method to determine NH_4_^+^.**

A potassium chloride solution is usually used to extract inorganic N from cattle manure. Tanahashi *et al.* (ref.**^8^**) found that cattle and swine manures contained the fraction of NH_4_^+^ that cannot be extracted by potassium chloride (ammonium magnesium phosphate; MAP) and examined 59 cattle manures (26 dairy, 28 beef, and 5 dairy and beef mix) and 52 swine manures made by various production methods for an appropriate extraction method of NH_4_^+^ containing MAP. They found that inorganic N containing MAP extracted by 0.5 mol L^-1^ hydrochloric acid in the condition of the 1 to 10 ratio of manure dry weight (g) and extract volume (mL) had the strongest relationship (R^2^ = 0.851, including some outliers, Fig. **S4b**) with N available from the manure in 4 weeks of incubation (30°C; 4-week N availability).

**Inorganic N and ADSN determinations for predicting short-term N availability.**

Tanahashi & Oyanagi (ref.**^5^**) conducted laboratory incubations (30°C, 84 days) for 59 composted cattle manures (26 dairy, 28 beef, and 5 dairy and beef mix) and 52 composted swine manures made by various production methods and determined ADOM and inorganic N in the samples. They found that (i) the relationship between the amount of N available from the applied compost in 28 days (4 weeks) of incubation (4-week N availability; Y_1_) and inorganic N in the compost (X) can be expressed as Y_1_ = X, regardless of the sample ADOM (Fig. **S5a**); however, for the relationship between the amount of N available from the applied compost in 84 days (12 weeks) of incubation (12-week N availability; Y_2_) and X (Fig. **S5b**), (ii) the Y_2_ = X relation continued for the samples that had < 250 mg g^-1^ of ADOM (95% of the composted cattle manure samples belonged to this category), whereas Y_2_ exceeded X for the samples that had ≥ 250 mg g^-1^ ADOM.

Oyanagi & Tanahashi (ref.**^6^**) conducted laboratory incubations (30°C, 84 days) for 79 composted cattle manures (42 dairy, 32 beef, and 5 dairy and beef mix), 71 composted swine manures, and 2 composted cattle and swine mix manures made by various production methods using the medium-to-coarse-grained brown lowland culture soil. N was determined by a dry combustion method using SUMIGRAPH NC-90A, Sumika Chemical Analysis Service, Japan. They found a positive linear relationship (Y = 0.50X – 2.5, R^2^ = 0.86) between 12-week N availability from the composted manure sample (Y) and ADSN in the sample (X) (Fig. **S6a**). This relationship was also confirmed for other types of culture soil with 116 composted manure samples (Fig. S6b).

**References**

1. Oyanagi, W., Ando, Y. & Tanahashi, T. Decomposition property of organic matters in soil and its indicators. *Japanese Journal of Soil Science and Plant Nutrition* **78(4)**, 407–410 (in Japanese) (2007).
2. Nishida, M. Nitrogen dynamics of organic materials applied to paddy fields: direct evaluation using organic materials labelled with nitrogen-15. *Japan Agricultural Research Quarterly* **45(1)**, 31–38 https://www.jstage.jst.go.jp/article/jarq/45/1/45_1_31/_pdf (2011).
3. Oyanagi, W., Tanahashi, T., Muramatsu, K. & Kobashi, Y. Utility of acid detergent soluble organism as index of decomposing organic materials easily. *Japanese Journal of Soil Science and Plant Nutrition* **81(4)**, 383–386 (in Japanese) (2010).
4. Oyanagi, W., Muramatsu, K. & Kobashi, Y. Recommendation of effective utilization of biomass and composts based on organic matter decomposition property. *Bulletin of the Niigata Animal Husbandry Experiment Station* **17**, 9–14 (in Japanese) (2011).
5. Tanahashi, T. & Oyanagi, W. Estimation of nitrogen fertilizer efficiency of cattle and swine manure composts based on acid-detergent-soluble organic matter and inorganic nitrogen. *Japanese Society of Soil Science and Plant Nutrition* **81(4)**, 336–342 (in Japanese with English summary) (2010).
6. Oyanagi, W. & Tanahashi, T. Estimation of nitrogen fertilizer effect on cattle compost and swine compost by acid detergent digestible nitrogen. *Japanese Journal of Soil Science and Plant Nutrition* **81(2)**, 144–147 (2010) (in Japanese).
7. Ando, Y., Oyanagi, W. & Moriyama, N. A simple method for the determination of nutrients content in organic matter using the small reflection photometer. *Japanese Society of Soil Science and Plant Nutrition* **75(5)**, 605–608 (in Japanese) (2004).
8. Tanahashi, T., Yano, H., Itou, H. & Oyanagi, W. Magnesium ammonium phosphate in cattle and swine manure composts and an extraction method for its evaluation. *Japanese Society of Soil Science and Plant Nutrition* **81(4)**, 329–335 (in Japanese with English summary) (2010).

**
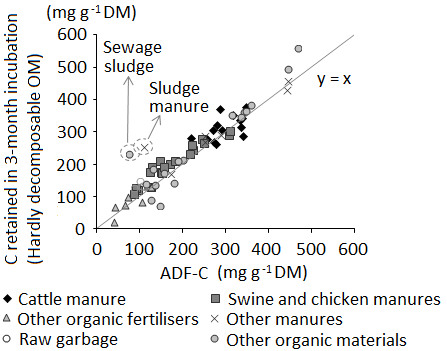
**

**Figure S1.** Relationship between C retained in 3 months of incubation in the soil and C contained in acid detergent fibre fraction (ADF-C) of the sample (quoted from Fig. 4 in Oyanagi *et al.* (ref.**^1^**). Two sludge-origin samples were found outliers, probably because polymer coagulant added for wastewater treatment**^1^**.

**
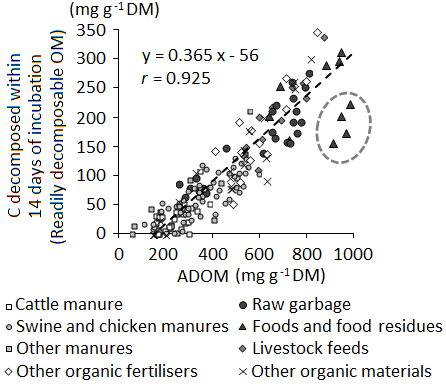

Figure S2.** Relationship between C decomposed within 14 days of incubation and acid detergent soluble organic matter (ADOM) of the sample (Fig. 2 in Oyanagi *et al.* (ref. **^3^**). The data enclosed by a dotted circle were high in starch, which were excluded from the regression analysis**^3^**.


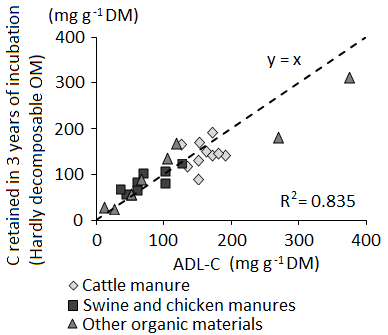


**Figure S3.** Relationship between C retained in 3 years of incubation in the soil and C contained in acid detergent fibre lignin (ADL-C) of the sample (Fig. 1 in Oyanagi *et al.* (ref.**^4^**).


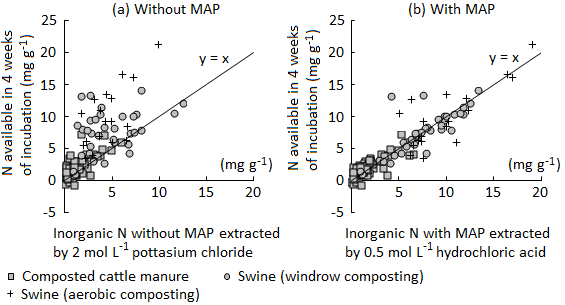


**Figure S4.** Relationships between (a) inorganic N without ammonium magnesium phosphate (MAP) extracted by 2 mol L^-1^ potassium chloride and (b) inorganic N with MAP extracted by 0.5 mol L^-1^ hydrochloric acid and N available in 4 weeks of incubation (Fig. 7 in Tanahashi *et al.* (ref.**^8^**)).

**
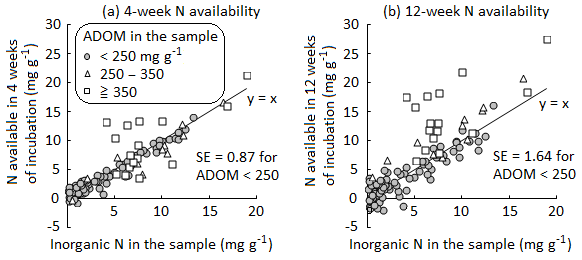
**

**Figure S5.** Relationships between (a) N available from the applied compost in 28 days (4 weeks) of incubation and inorganic N in the compost (Fig. 4 in Tanahashi & Oyanagi (ref.**^5^**) and (b) N available from the compost in 84 days (12 weeks) of incubation and inorganic N in the sample (Fig. 5 in Tanahashi & Oyanagi (ref.**^5^**); the figures were rearranged by the size of ADOM; rearranged by Tanahashi).


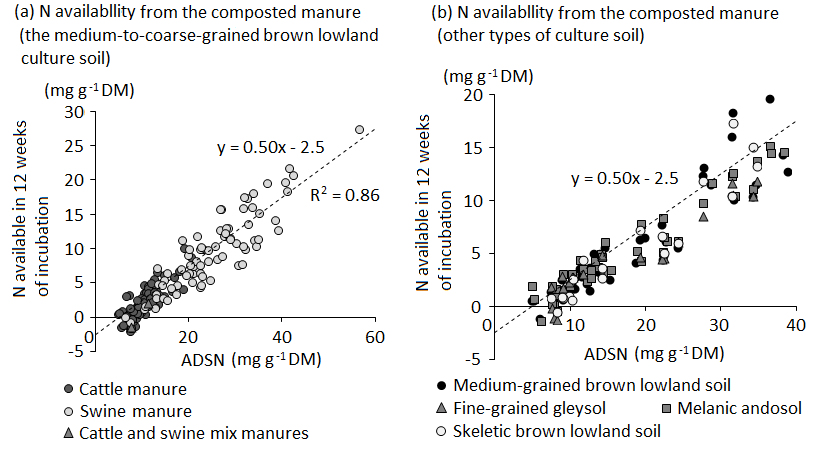


**Figure S6.** (a) Relationship between N available from the applied manure in 84 days (12 weeks) of incubation and acid detergent soluble nitrogen (ADSN) in the manure (a medium-to-coarse-grained brown lowland soil was used as a culture soil; quoted from Fig. 2 in Oyanagi & Tanahashi (ref.**^6^**). (b) This relationship when other types of culture soils were used (quoted from Fig. 3 in Oyanagi & Tanahashi (ref.**^6^**)).
